# Unraveling the H19/GAS1 axis in recurrent implantation failure: A potential biomarker for diagnosis and insight into immune microenvironment alteration

**DOI:** 10.1371/journal.pone.0306244

**Published:** 2024-07-05

**Authors:** Li Fan, Fan Zhang, Chunling Yao, Liuying Nong, Jingjing Li, Wenjie Huang

**Affiliations:** 1 Department of Reproductive Medicine, Guangzhou Women and Children’s Medical Center Liuzhou Hospital, Liuzhou, Guangxi, China; 2 Reproductive Medicine Center, Liuzhou Maternity and Child Health Care Hospital, Liuzhou, China; 3 Guangxi Maternal and Obstetric Disease Research Center, Liuzhou, China; 4 Liuzhou Institute of Reproduction and Genetics, Liuzhou Maternity and Child Health Care Hospital, Liuzhou, China; Tokyo University of Pharmacy and Life Sciences: Tokyo Yakka Daigaku, JAPAN

## Abstract

Recurrent implantation failure (RIF) presents a significant clinical challenge due to the lack of established diagnostic and therapeutic guidelines. Emerging evidence underscores the crucial role of competitive endogenous RNA (ceRNA) regulatory networks in non-cancerous female reproductive disorders, yet the intricacies and operational characteristics of these networks in RIF are not fully understood. This study aims to demystify the ceRNA regulatory network and identify potential biomarkers for its diagnosis. We analyzed expression profiles of three RNA types (long noncoding RNAs [lncRNAs], microRNAs [miRNAs], and mRNAs) sourced from the GEO database, leading to the identification of the H19-hsa-miR-301a-3p-GAS1 ceRNA network. This network demonstrates significant diagnostic relevance for RIF. Notably, the H19/GAS1 axis within this ceRNA network, identified through correlation analysis, emerged as a promising diagnostic marker, as evidenced by operating receiver operator characteristic (ROC) curve analysis. Further investigation into the binding potential of miR-301a-3p with H19 and GAS1 revealed a close association of these genes with endometrial disorders and embryo loss, as per the Comparative Toxicogenomics Database. Additionally, our immune infiltration analysis revealed a lower proportion of T cells gamma delta (γδ) in RIF, along with distinct differences in the expression of immune cell type-specific markers between fertile patients and those with RIF. We also observed a correlation between aberrant expression of H19/GAS1 and these immune markers, suggesting that the H19/GAS1 axis might play a role in modifying the immune microenvironment, contributing to the pathogenesis of RIF. In conclusion, the ceRNA-based H19/GAS1 axis holds promise as a novel diagnostic biomarker for RIF, potentially enhancing our understanding of its underlying mechanisms and improving the success rates of implantation.

## Introduction

Since the landmark birth via in vitro fertilization (IVF) over four decades ago, the global utilization of assisted reproductive technologies has notably increased. Despite the progressive improvements in IVF success rates [1], a significant number of embryo transfers still fail to result in implantation. In recent years, there has been a growing focus on understanding implantation failure, particularly Recurrent Implantation Failure (RIF). However, the lack of a uniform consensus on defining RIF highlights the complexity of its etiology, which encompasses paternal, maternal, and embryonic factors [2]. Among these, the maternal factor, particularly the endometrium’s receptivity, is crucial for successful embryo implantation and the ensuing pregnancy.

It is widely acknowledged that endometrial receptivity is a multifaceted phenomenon, governed by a spectrum of physiological and molecular mechanisms. Although recent studies have identified potential biomarkers of endometrial receptivity in RIF, including leukemia inhibitor factor [3], protein 53 [4], human leukocyte antigen [5], vascular endothelial growth factor [6], there remains a substantial gap in our understanding of the detailed molecular mechanisms at play. This gap underscores the need for deeper research into the molecular dynamics of the endometrium during the implantation window. Furthermore, in humans, certain alterations in uterine decidual macrophages [7], uNK cell functionality [8], and cytokine levels [9] are linked to improper trophoblast invasion and implantation failure. Shifts in CD4+ and CD8+ T lymphocytes and B cell counts [10] are associated with recurrent miscarriage and RIF, underscoring the pivotal role of immune dysregulation in these reproductive challenges.

The burgeoning interest in RNA biology has significantly illuminated the multifaceted regulatory roles of noncoding RNA in recent years [11]. MicroRNAs (miRNAs), a subset of small, endogenous, nonprotein-coding RNAs, are recognized for their conservation across species and their pivotal role in posttranscriptional gene regulation in eukaryotic cells [12]. Remarkably, it is estimated that miRNAs mediate control over roughly 30% of all protein-coding genes in the human genome [13]. Recent research has increasingly highlighted the critical role of miRNAs in RIF [14, 15] and embryo development [16, 17], pointing towards their potential as diagnostic biomarkers for RIF.

Long noncoding RNAs (lncRNAs), defined as noncoding RNA molecules exceeding 200 nucleotides in length, have been shown to engage in a variety of biological processes. These include gene expression regulation, genetic imprinting, histone modification, and chromatin dynamics [18]. Over a decade ago, Salmena et al. [19] introduced the concept of competitive endogenous RNA (ceRNA), positing that noncoding RNAs and mRNAs compete for miRNA response elements (MREs). This concept underscores the regulatory influence of lncRNAs on mRNAs via a ceRNA mechanism.

While such regulatory functions have been extensively explored in cancer biology, their implications in RIF remain less explored [20]. In light of this, our study aims to explore the involvement of the ceRNA network in regulating uterine receptivity for embryo implantation. In this context, our interest has been particularly drawn to H19 and GAS1, two genes that have emerged from our preliminary bioinformatics analysis as significantly dysregulated in RIF compared to normal endometrial tissue. H19, a well-known lncRNA, and GAS1, a gene implicated in cell growth arrest, have been hypothesized to interact through the hsa-miR-301a pathway, based on our computational predictions. By investigating this network, we seek to deepen our understanding of the molecular interplay at the interface of implantation biology and RNA regulation.

## Materials and methods

### Data preparation and processing

We downloaded the gene expression profiles (GSE111974, including 24 fertile control samples and 24 RIF samples at the window of implantation; GPL17077) and the miRNA expression profiles (GSE71332, including 5 fertile control samples and 7 RIF samples at the window of implantation; GPL18402) from the GEO database (http://www.ncbi.nlm.nih.gov/geo). Meanwhile, GSE26787 (including 5 fertile control samples and 5 RIF samples at the middle luteal phase; GPL570) was used to further validate our results. The series matrix file was then converted from gene probe IDs to gene symbol codes. When different probes corresponded to the same gene, the mean expression value was taken as the gene expression value. The raw data were quantile normalized and log2-transformed prior to analysis.

### Screening of differentially expressed genes

For differential gene expression (DEG) analysis, we determined the DElncRNAs, DEmiRNAs, and DEmRNAs with thresholds of |logFC| > 1.0 and *p* < 0.05. Volcano plots of DERNAs (including DElncRNAs, DEmiRNAs, and DEmRNAs) and a heat map were drawn using the ggplot2 and ComplexHeatmap packages of the R software (version 4.2.1) [21]. Genes were only included if the standard nomenclature identified them as human genes in the HGNC database. In the initial screening of DEGs, we employed an unadjusted p-value threshold of <0.05 to broadly identify candidates for further analysis. This approach was intended to maximize the discovery potential geven the complex etiology of RIF. Subsequently, to ensure the statistical rigor of our findings, we applied false discovery rate (FDR) adjustments to the identified candidates.

### Establishment of the ceRNA network in RIF

According to this hypothesis, lncRNA can act as a miRNA natural sponge in the cytoplasm and can indirectly regulate mRNA expression. This was established based on the following steps. First, we used miRNet (https://www.mirnet.ca/) to predict the potential target miRNA of lncRNA and the lncRNA-miRNA interaction pairs. Then, the DEmiRNA target genes were predicted using miRTarBase [22], TarBase [23], and miRecords [24], and the miRNA-mRNA interaction pairs were built. A Venn diagram was used to visualize the DEmiRNA that overlapped with lncRNA-target miRNA (DETmiRNAs), and DEmRNAs that overlapped with DETmiRNA-target mRNA, respectively. Finally, we built the lncRNA-miRNA-mRNA triple regulatory network based on the lncRNA-miRNA pairs and miRNA-mRNA pairs.

DElncRNA sequencing data were extracted from LNCipedia (https://lncipedia.org/), and LncLocator (http://www.csbio.sjtu.edu.cn/bioinf/lncLocator/) was applied to predict the cellular location according to its sequence. The lncRNA-miRNA-mRNA interaction network was visualized using the Cytoscape software (https://www.cytoscape.org/), and the CytoHubba plugin in Cytoscape was used to identify the hub triple regulatory network.

The Comparative Toxicogenomics Database (CTD, http://ctdbase.org/) is a digital resource that facilitates the discovery of novel associations between chemicals and health outcomes through molecular mechanisms. We used this database to query the interacting chemicals for H19 and GAS1 and explored genes with a high degree of similarity to H19 and GAS1 from the perspective of common interacting chemicals.

The correlations among genes were analyzed by Pearson correlation analysis, and the plots were performed with the R package ggplot. The absolute value of a correlation coefficient above 0.8 was considered a high degree of correlation; a correlation coefficient from 0.5–0.8 represented a moderate correlation, while a correlation coefficient ranging from 0.3–0.5 indicated a low degree of correlation.

Receiver operating characteristic (ROC) curve analyses were performed using the R package pROC. An area under the curve (AUC) value from 0.5–0.7 indicated a lower predictive value, an AUC from 0.7–0.9 indicated a moderate predictive value, and an AUC from 0.9–1.0 indicated a high predictive value.

### Functional enrichment analysis

To better understand the possible biological processes and pathways of the network, we conducted a functional enrichment analysis of the enrichment of pathways and biological processes. We used the clusterProfiler package, and the GOplot package was utilized to calculate the z-score values corresponding to each enriched term based on the provided numerical values of molecule. In addition, we used the STRING website (https://string-db.org/) to obtain the top 40 GAS1-binding proteins, and KEGG and GO enrichment (including BP, CC, and MF) analyses of these genes were performed using the R packages clusterProfiler and msigdbr. The results were visualized with the ggplot2 R package. An adjusted *p* value of less than 0.05 was considered statistically significant.

### Evaluation of altered immune cell types

In this study, we initially identified 24 immune markers previously reported in the literature [25]. Subsequently, we employed single-sample Gene Set Enrichment Analysis (ssGSEA) based on the gene set variation analysis (GSVA) algorithm to calculate the abundance of these immune cell types in endometrial samples. CIBERSORT, another method for estimating cell abundance from expression profiles, was utilized in our research. We employed CIBERSORT to assess the relative proportions of 22 immune cell subpopulations, followed by Wilcoxon tests to determine the significant differences in immune cell types between the fertile control group and the RIF group, between the H19 high-expression and H19 low-expression groups, and between the GAS1 high-expression and GAS1 low-expression groups. They were divided into high- and low-expression groups according to median gene expression. Additionally, the stromal and immune scores for both groups were computed using an algorithm provided by the R package "estimate", referencing biomarkers supplied by a previously reported study [26].

### Statistical analysis

In this study, all data analysis and visualizations were performed using the R software (version 4.2.1; **https://www.r-project.org/**) with the appropriate package. The Wilcoxon test applied to identify differentially expressed genes, considering its suitability for non-normally distributed data. A p-value of less than 0.05 was deemed statistically significant. For correlation analyses between gene expression, Pearson’s correlation coefficient was used to assess the strength and direction of linear relationships.

## Results

### Differentially Expressed lncRNAs, miRNAs, and mRNAs in RIF

**Fig 1:**
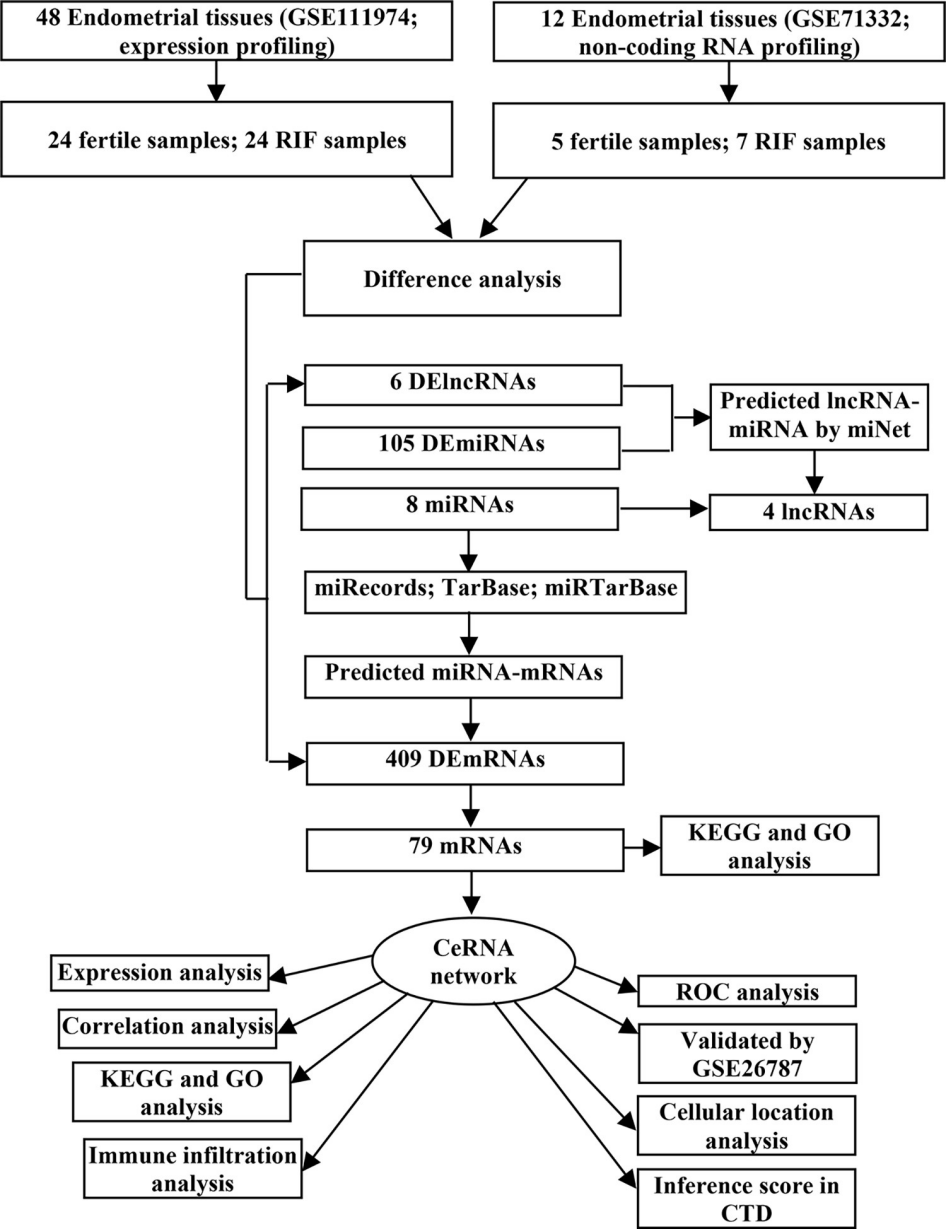
Flowchart of construction and analysis ceRNA.

**Fig 2:**
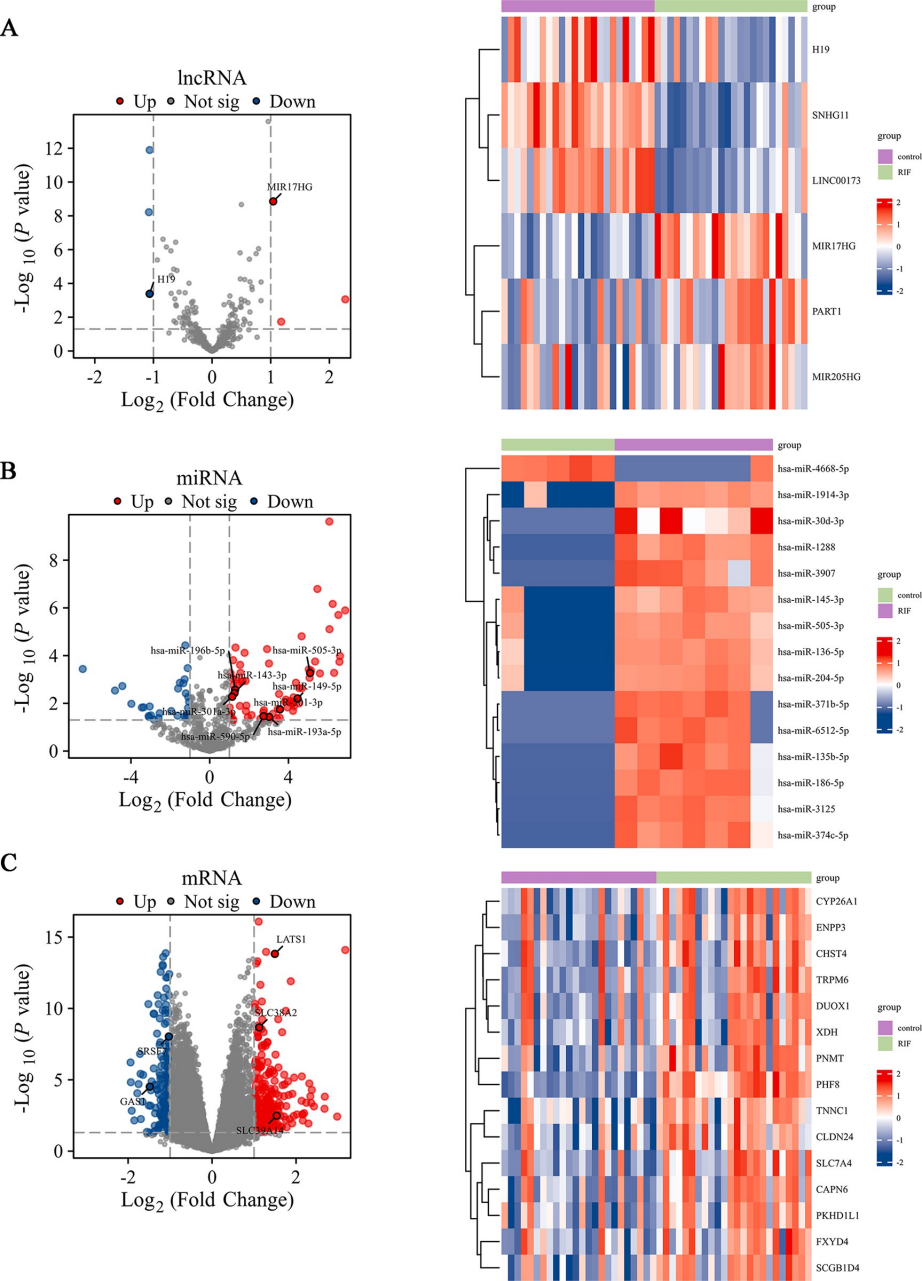
Volcano plots and heatmap plots of DElncRNAs, DEmiRNAs, and DEmRNAs between fertile and recurrent implantation failure groups during the window of implantation. (A-C) The volcano plots and horizontal axis of the heatmap (15 significant DEGs) describe DElncRNA (A), DEmiRNA (B), and DEmRNA (C) (|log_2_fold change| > 1 and *p*-value < 0.05).

Fig 1 shows the workflow of this study. We first identified the DElncRNAs, DEmiRNAs, and DEmRNAs in RIF and fertile endometrial samples using the GEO database, with |log fold change (FC)| > 1.0 and *p* < 0.05 as the threshold. Based on the HGNC database, a total of 6 DElncRNAs (3 upregulated and 3 downregulated), 105 DEmiRNAs (75 upregulated and 30 downregulated), and 409 DEmRNAs (237 upregulated and 172 downregulated) were detected from RIF samples and fertile endometrial samples. Volcano plots illustrate the distribution of DElncRNAs (Fig 2A), DEmiRNAs (Fig 2B), and DEmRNAs (Fig 2C), and heatmaps depict the expression of the top variable genes in RIF and fertile samples (Fig 2A–2C).

### Establishing the lncRNA-miRNA-mRNA triple regulatory network for RIF

**Fig 3:**
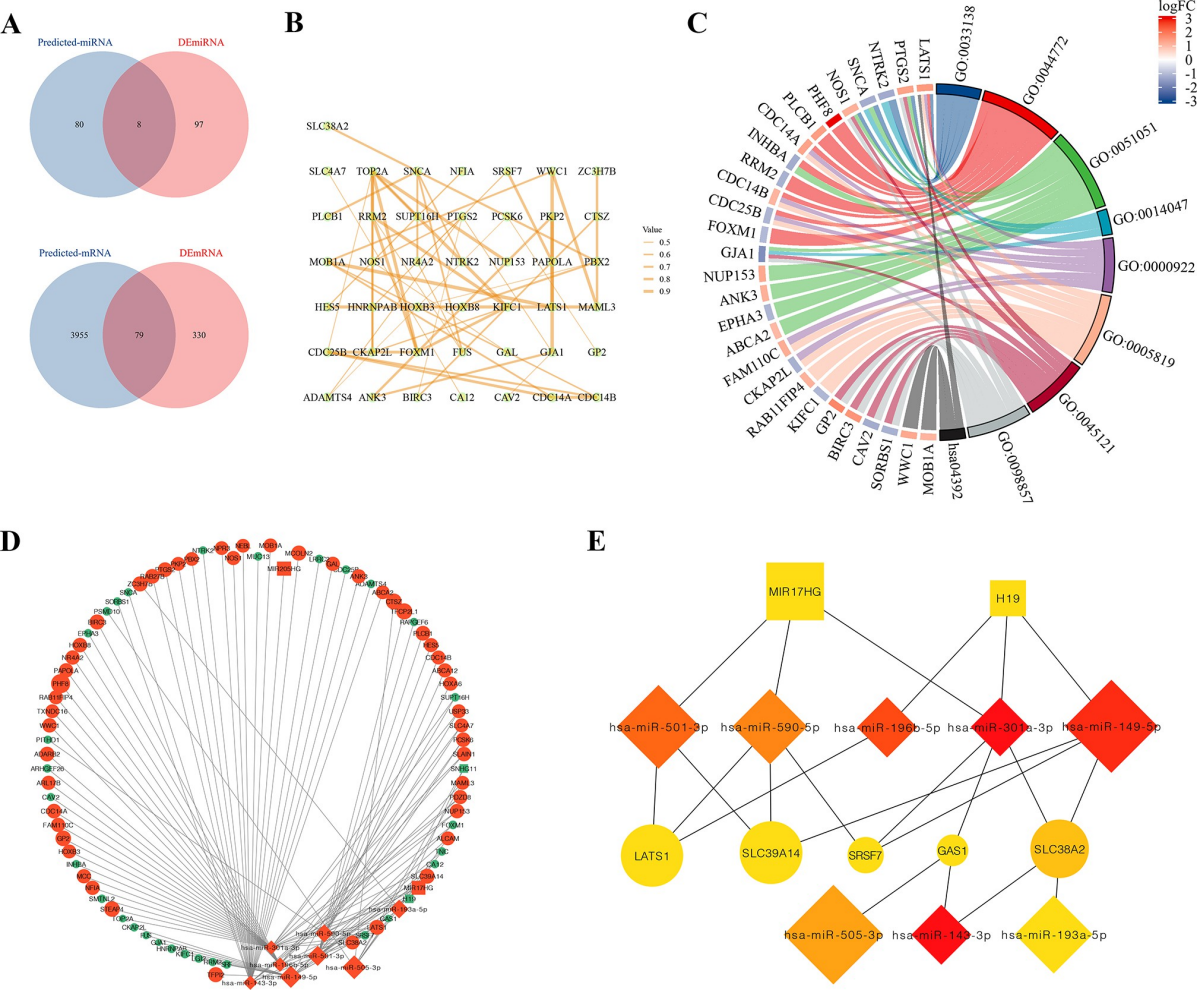
Construction and functional and enrichment analysis of the lnRNA-miRNA-mRNA triple regulatory network in RIF. (A) Intersection analysis using Venn diagrams to identify the overlap between predicted DElnRNAs-target miRNAs and the DEmiRNAs, as well as the overlap between predicted common miRNA-target mRNAs and DEmRNAs. (B) Protein-Protein Interaction (PPI) network constructed using the STRING database. (C) Functional enrichment analysis, including both KEGG and GO, was performed on the intersection intersected mRNAs. (D) Illustration of the triple regulatory network in RIF, with genes marked in red indicating upregulation and those in green representing doenregulation. (E) Fifteen hub genes in this network with a score of >3.

To obtain the lncRNA-miRNA-mRNA triple regulatory network in RIF, we performed a joint analysis in RIF and the fertile endometrial tissue groups. First, we entered the six DElncRNAs into the miRNet database to identify potential miRNAs targeting lncRNAs. Of the 88 predicted miRNAs, 8 were selected after taking the intersection with DEmiRNAs (Fig 3A). We then used the miRNet databases to identify the downstream target mRNAs with reference to the 8 DEmiRNAs, and 79 mRNAs were identified according to the intersection of 409 DEmRNAs and 4034 predicted mRNAs (Fig 3A). To investigate the interactions between common differential genes, we employed the String online database to construct the PPI network, incorporating the 79 identified genes. Subsequently, we refined the PPI network by removing genes that lacked interactions, resulting in a network containing 43 nodes and 51 edges (Fig 3B). Moreover, to delve deeper into the potential involvement of the 79 genes in embryo implantation, we performed a comprehensive functional enrichment analysis, encompassing both KEGG and GO annotations. The outcomes of the analysis revealed that all 79 genes exhibited significant enrichment in pivotal biological processes, notably the cell cycle, membrane raft, and hippo signaling pathway, which is probably related to the preimplantation, implantation, and postimplantation developmental stages (Fig 3C and Table 1). Finally, a total of 3 lncRNAs (2 upregulated and 2 downregulated), 8 miRNAs (upregulated), and 79 mRNAs (49 upregulated and 30 downregulated) were included to construct the RIF-associated lncRNA-miRNA-mRNA triple regulatory network using the Cytoscape software (Fig 3D). Furthermore, we used the Cytoscape plugin cytoHubba to find the hub regulatory network. Our results showed that two lncRNAs (H19 and MIR17HG), eight miRNAs (miR-501-3p, miR-590-5p, miR-196b-5p, miR-301a-3p, miR-149-5p, miR-505-3p, miR143-3p, and miR-193a-5p), and five mRNAs (LATS1, SLC39A14, SRSF7, GAS1, and SLC38A2) were identified (Fig 3E).

**Table 1:** Top 5 significantly GO terms and KEGG pathways.

Ontology	ID	Description	GeneRatio	BgRatio	p.adjust
BP	GO:0048568	embryonic organ development	11/40	449/18800	1.3891E-07
BP	GO:0048566	embryonic digestive tract development	4/40	33/18800	1.8382E-05
BP	GO:0016331	morphogenesis of embryonic epithelium	6/40	150/18800	1.8621E-05
BP	GO:0048562	embryonic organ morphogenesis	7/40	294/18800	4.9556E-05
BP	GO:0030326	embryonic limb morphogenesis	5/40	119/18800	8.7927E-05
CC	GO:0097542	ciliary tip	5/40	47/19594	6.42e-06
CC	GO:0097546	ciliary base	4/40	42/19594	0.0001
CC	GO:0045121	membrane raft	6/40	326/19594	0.0020
CC	GO:0098857	membrane microdomain	6/40	327/19594	0.0020
CC	GO:0060170	ciliary membrane	3/40	75/19594	0.0157
MF	GO:1990841	promoter-specific chromatin binding	3/40	62/18410	0.0310
MF	GO:0002039	p53 binding	3/40	66/18410	0.0310
MF	GO:0030971	receptor tyrosine kinase binding	3/40	76/18410	0.0313
MF	GO:0008013	beta-catenin binding	3/40	86/18410	0.0337
MF	GO:0035035	histone acetyltransferase binding	2/40	23/18410	0.0355
KEGG	hsa04340	Hedgehog signaling pathway	18/28	56/8164	5.23e-32
KEGG	hsa05217	Basal cell carcinoma	10/28	63/8164	1.63e-13
KEGG	hsa04360	Axon guidance	5/28	182/8164	0.0088
KEGG	hsa05205	Proteoglycans in cancer	5/28	205/8164	0.0114
KEGG	hsa04024	cAMP signaling pathway	4/28	221/8164	0.0912

Furthermore, all genes identified through our screening process exhibited adjusted p-value <0.05, underscoring their statistical significance in the context of our study.

### Construction and validation of the ceRNA network and selection of a model with an RIF-specific diagnostic value

**Fig 4:**
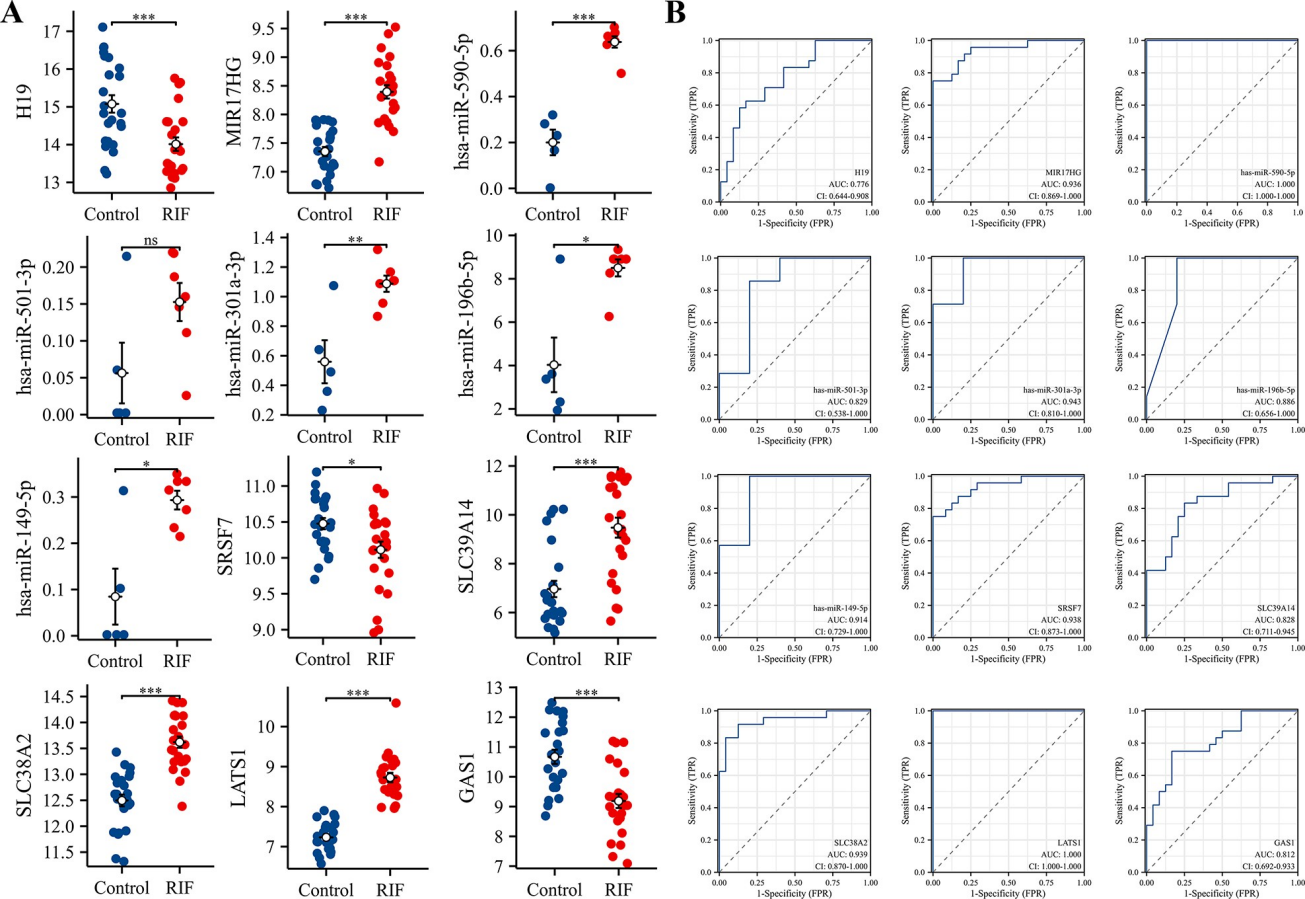
Expression patterns and ROC curve analysis of the 12 hub-RNA between fertile and RIF groups. (A) Depicts the expression patterns of two hub-DElnRNAs, five hub-DEmiRNA, and five hub-DEmRNAs in RIF compared to fertile endometrial tissues. (B) ROC curve analysis of these 12 hub-genes in RIF samples. ROC, receiver operating characteristic curves. AUC, area under the ROC curve. *p < 0.05; **p < 0.01; ***p < 0.001; ns, no significant difference was observed.

To establish a crucial ceRNA of great diagnostic value, we first analyzed the expression levels of RNAs from the hub triple regulatory network in RIF and fertile endometrial tissues. As showcased in Fig 4A, we found one downregulated (H19) and one upregulated (MIR17HG) lncRNA, five upregulated (miR-590-5p, miR-196b-5p, miR-301a-3p, miR-149-5p, miR-505-3p, miR143-3p, and miR-193a-5p) and one undifferentiated (miR-501-3p) miRNA, three upregulated (LATS1, SLC39A14, and SLC38A2) and two downregulated (SRSF7 and GAS1) mRNAs in RIF.

To further assess the potential diagnostic role of these RNAs in RIF, ROC curve analysis was performed. In total, H19, miR-501-3p, miR-196b-5p, SLC39A14, and GAS1 had moderate predictive value (AUC > 0.7), whereas MIR17HG, miR-509-5p, miR-301a-3p, miR-149-5p, SRSF7, SLC38A2, and LATS1 (AUC > 0.9) had a high predictive value (Fig 4B).

**Fig 5:**
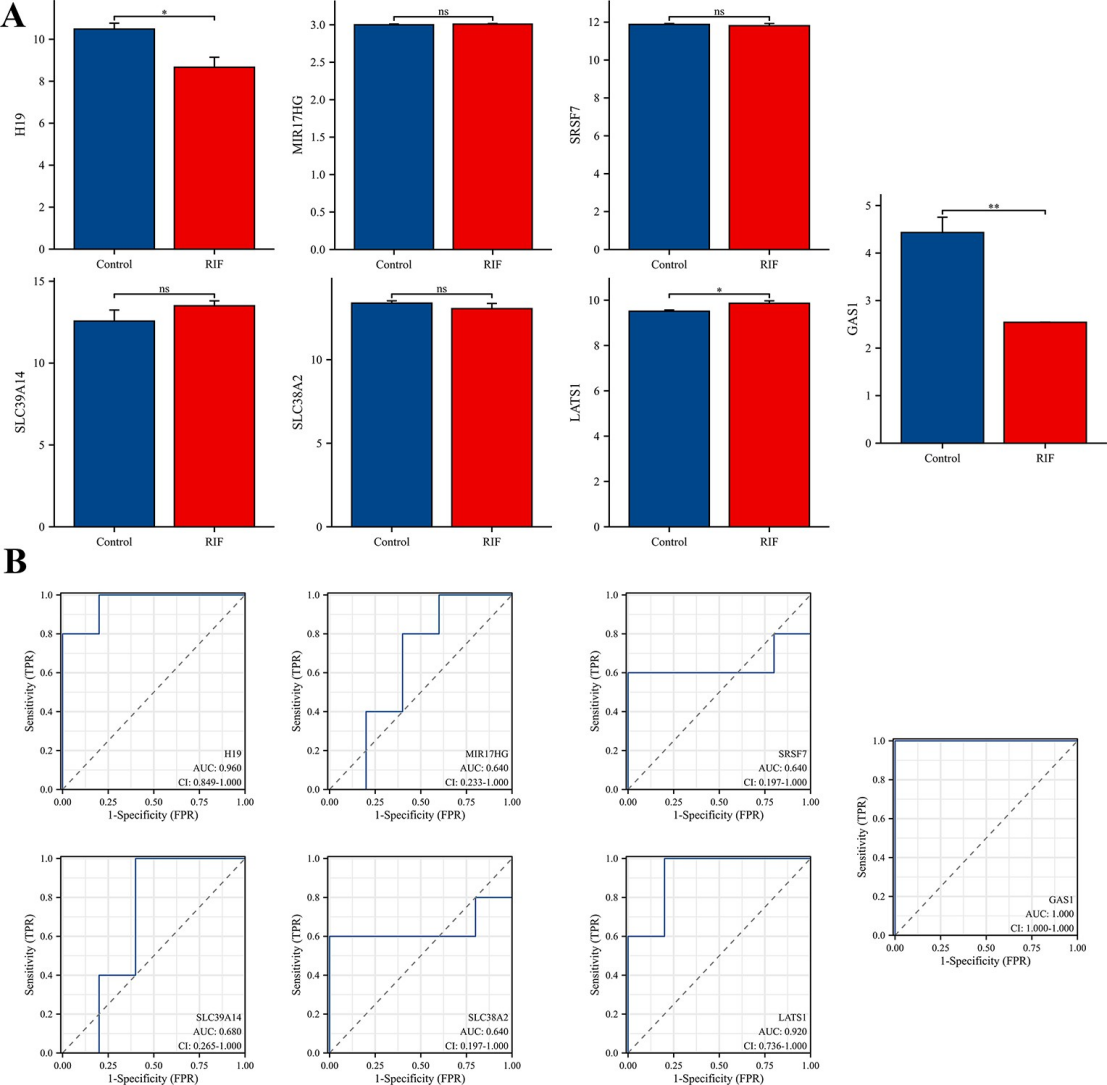
Validation of hub genes and ROC curve analysis in GSE26787. (A-B) Analysis of expression patterns and ROC curves for key differentially expressed two hub-DElnRNAs and five hub-DEmRNAs in RIF copmpared to ertile endometrial tissues. *p < 0.05; **p < 0.01; ns, no significant difference was observed.

To confirm the accuracy of the obtained results, we used another dataset, GSE26787, as a validating dataset. Our results showed that H19 and GAS1 were significantly downregulated while LATS1 was significantly upregulated in RIF patients. No significant differences in MIR17HG, SRSF7, SLC39A14, or SLC38A2 were observed (Fig 5A). Similarly, the ROC analysis showed that H19, GAS1, and LATS1 had a high predictive value (AUC > 0.9), while MIR17HG, SRSF7, SLC39A14, and SLC38A2 had a lower predictive value (AUC < 0.7; Fig 5B). Only one lncRNA and two mRNAs were consistent in the training dataset GSE111974 and the validating dataset GSE26787.

**Fig 6:**
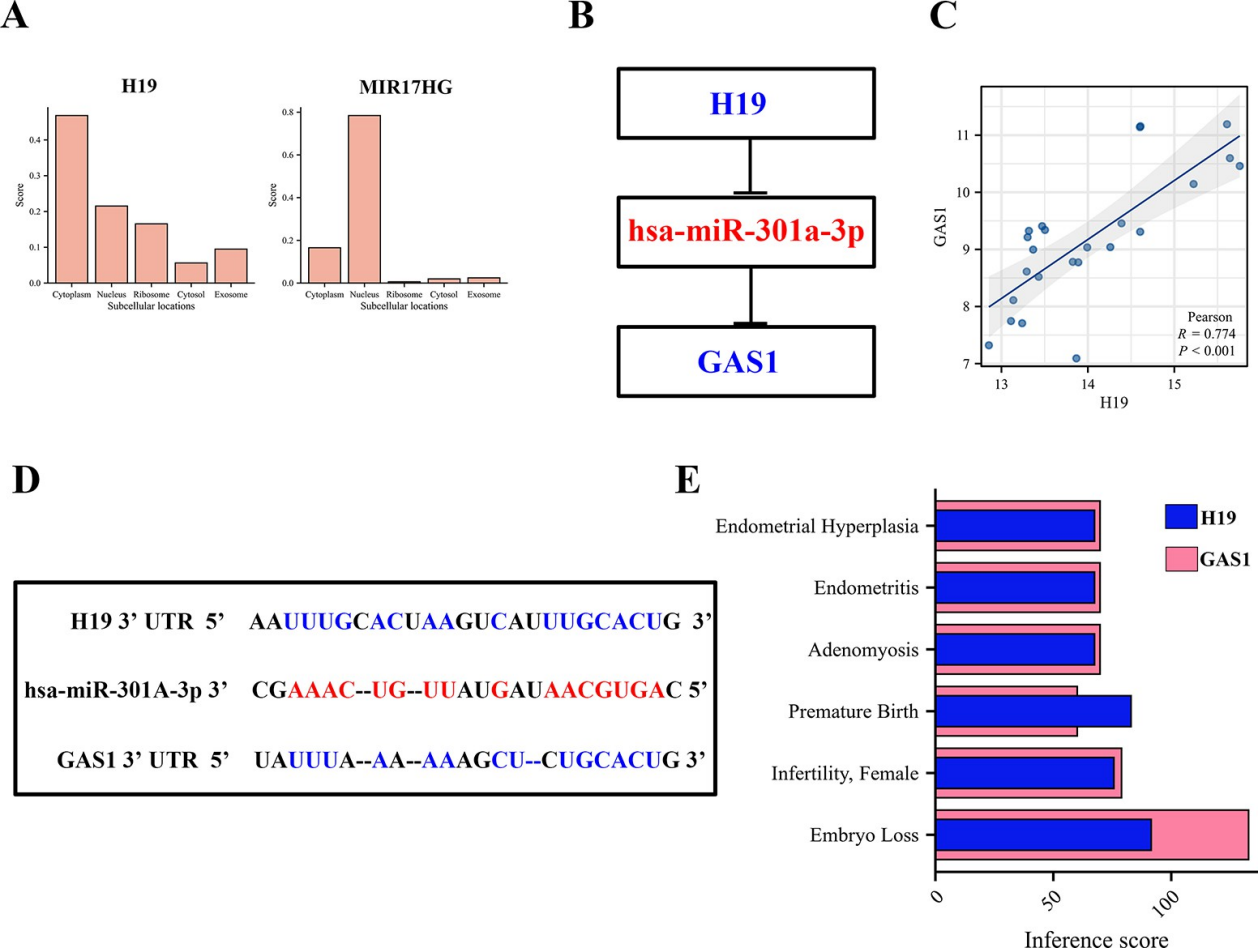
Construction and correlation analysis of the ceRNA network. (A) Prediction of cellular localization for two key lncRNAs (H19 and MIR17HG) using the lncLocator tool. (B) Schematic representation of the ceRNA network. Genes upregulated in this network are marked in red, while downregulated genes are indicated in blue, visually depicting their regulatory dynamics. (C) Correlation analysis exploring the relationship between H19 and GAS1 in RIF, highlight the potential interaction between these two critical genes. (D) Base pairing prediction between miR-301a-3p and target site in the 3’ UTR of H19 and GAS1, as forecasted by ENCORI database. (E) Analysis of the correlation between hub genes and uterine disorders using Comparative Toxicomics Database, showcasing the broader implication of these genes in gynecological pathologies.

Considering that the cellular localization of lncRNA is closely related to its molecular mechanism, we analyzed the subcellular localization of H19 and MIR17HG using lncLocator. As shown in Fig 6A, H19 was mainly located in the cytoplasm, while MIR17HG was mainly distributed in the nucleus. In addition, the expression correlation analysis indicated a positive relationship between H19 and GAS1 expression in RIF (Fig 6C). These data indicate that H19 may act as a ceRNA to improve the expression of GAS1 by sponging miR-301a-3p. Thus, we constructed an H19-miR-301a-3p-GAS1 ceRNA network (Fig 6B). The target sites in the H19 and GAS1 3’ UTRs were predicted to pair with miR-301a-3p using the ENCORI database (Fig 6D). The CTD database shows that H19 and GAS1 are associated with uterine disorders, especially embryo loss (Fig 6E). Furthermore, data from the CTD listed 24 chemicals related to H19 and GAS1. Specifically, eight chemicals were identified to upregulate H19, and two to upregulate GAS1. Conversely, twelve chemicals could downregulate 19, and eighteen could downregulate GAS1. Additionally, four chemicals have been verified to affect the expression of H19 and GAS1, although their exact mechanisms of action remain unclear (Table 2). Moreover, through chemicals association studies, we identified the top 20 relationships between H19, GAS1, and other genes. The findings indicated a high correlation of H19 with cathepsin H (CTSH), RAS guanyl releasing protein 2 (RASGRP2), and thiol methyltransferase 1A (TMT1A). GAS1 showed a significant correlation with spectrin repeat containing nuclear envelope protein 1 (SYNE1), E74 like ETS transcription factor 3 (ELF3), myelin protein zero like 2 (MPZL2), and RAB GTPase activating protein 1 like (RABGAP1L) (Table 3). Thus, the H19/GAS1 axis in the ceRNA network was selected as a potential diagnostic model for the following step analysis.

**Table 2:** Interacting chemicals of H19 and GAS1 in CTD.

Chemical name (H19)	ID	Interaction actions	Chemical name (GAS1)	ID	Interaction actions
1-Methyl-3isobutylxanthine	D015056	increases expression	1,4-bis(2-(3,5-dichloropyridyloxy))benzene	C028474	increases expression
2-(1’H-indole-3’-carbonyl)thiazole-4-carboxylic acid methyl ester	C548651	increases expression	1-Butanol	D020001	decreases expression
2-amino-4-phosphonobutyric acid	C012729	increases expression	1-nitropyrene	C032668	decreases expression
2-methyl-2H-pyrazole-3-carboxylic acid (2-methyl-4-o-tolylazophenyl)amide	C511621	decreases expression	2-butenal	C012796	decreases expression
7,8-Dihydro-7,8-dihydroxybenzo(a)pyrene 9,10-oxide	D015123	decreases expression	3,4,5,3’,4’-pentachlorobiphenyl	C023035	decreases expression
9,10-Dimethyl-1,2-benzanthracene	D015127	affects expression	4,4’-diaminodiphenylmethane	C009505	increases expression
acetamide	C030686	increases expression	4,4’-hexafluorisopropylidene diphenol	C583074	decreases expression
Acetaminophen	D000082	decreases expression	4-(5-benzo(1,3)dioxol-5-yl-4-pyridin-2-yl-1H-imidazol-2-yl)benzamide	C459179	decreases expression
Acrylamide	D020106	decreases expression	7,8-Dihydro-7,8-dihydroxybenzo(a)pyrene 9,10-oxide	D015123	decreases expression
Aflatoxin B1	D016604	decreases expression	Acetaldehyde	D000079	affects expression
Air Pollutants	D000393	affects expression	Acetaminophen	D000082	affects expression
Aldehydes	D000447	decreases expression	Acrylamide	D020106	decreases expression
Antirheumatic Agents	D018501	increases expression	Aflatoxin B1	D016604	decreases expression
Arsenic	D001151	decreases expression	Air Pollutants	D000393	decreases expression
Arsenicals	D001152	decreases expression	Arsenic	D001151	decreases expression
arsenite	C015001	increases expression	Arsenicals	D001152	decreases expression
Asbestos, Serpentine	D017632	decreases expression	Arsenic Trioxide	D000077237	decreases expression
Benzo(a)pyrene	D001564	decreases expression	benz(a)anthracene	C030935	decreases expression
benzyloxycarbonylleucyl-leucyl-leucine aldehyde	C072553	decreases expression	Benzo(a)pyrene	D001564	decreases expression
beta-methylcholine	C044887	affects expression	beta-methylcholine	C044887	affects expression
bis(4-hydroxyphenyl)sulfone	C543008	increases expression	beta-Naphthoflavone	D019324	decreases expression
bisphenol A	C006780	affects expression	bis(4-hydroxyphenyl)sulfone	C543008	decreases expression
bisphenol F	C000611646	increases expression	bisphenol A	C006780	affects expression
butylbenzyl phthalate	C027561	decreases expression	bisphenol F	C000611646	decreases expression

**Table 3:** Relationship of H19 and GAS1 with genes via chemical interaction, based on the CTD database.

Gene (H19)	Similarity	Common interacting chemicals	Gene (GAS1)	Similarity	Common interacting chemicals
CTSH	0.3058	63	SYNE1	0.3056	55
RASGRP2	0.3043	49	ELF3	0.3043	56
TMT1A	0.3029	53	MPZL2	0.3006	49
KRT23	0.2989	55	RABGAP1L	0.3000	51
ARHGAP22	0.2988	49	JADE1	0.2989	52
SYNM	0.2983	54	PRICKLE1	0.2989	52
CD52	0.2978	53	DUSP10	0.2947	61
NTN1	0.2965	51	CENPA	0.2878	59
PLXDC2	0.2965	51	GRHL1	0.2866	47
C1QTNF6	0.2919	47	SPRY1	0.2841	50
CRISPLD2	0.2915	58	ANTXR1	0.2833	51
CRABP1	0.2909	48	KRT23	0.2833	51
RBP1	0.2883	54	SCARA3	0.2831	47
TMEM158	0.2874	50	NUDT7	0.2825	50
COTL1	0.2871	50	ANKRD1	0.2814	56
RERG	0.2866	45	ETV5	0.2813	54
NDRG4	0.2857	54	SMOC1	0.2802	51
CARD10	0.2840	48	EPDR1	0.2789	41
TCEAL8	0.2830	45	ZBTB16	0.2778	60
EFNB2	0.2827	54	CRISPLD2	0.2769	54

### Immune cell composition analysis in RIF

**Fig 7:**
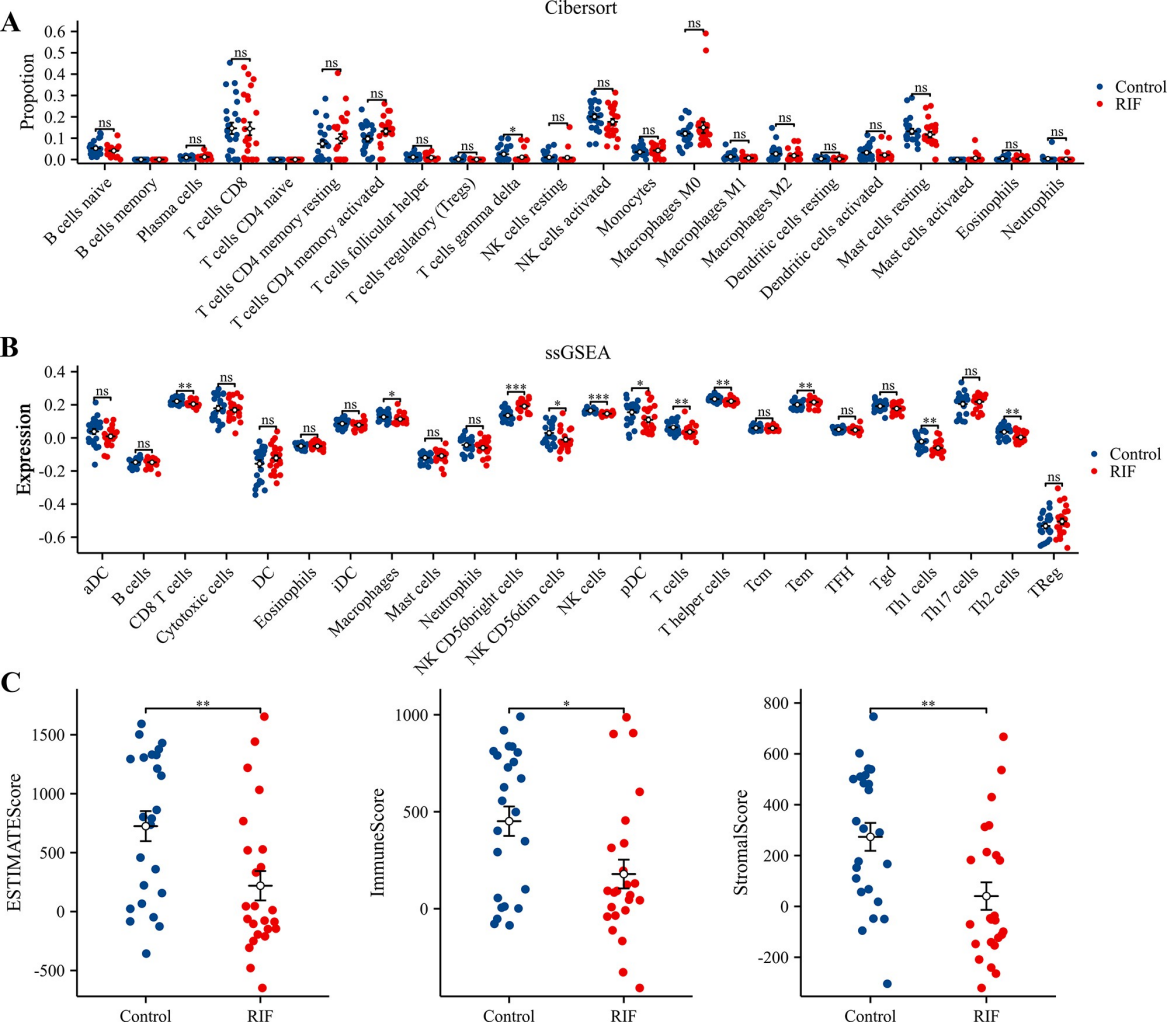
Immune cell infiltration in RIF patients. (A) Proportional distribution of 22 distinct immune cell types comparing fertile and RIF patient groups, illustrating the immune cell composition in each group. (B) Expression analysis of specific markers for immune cell types in both groups, highlighting the differential marker expression between fertile and RIF patients. (C) Comparative analysis of StromalScore, ImmuneScore, and EstimateScore between fertile and RIF patients, indicating various in the stromal and immune landscape within the endometrial microenvironment. *p < 0.05; **p < 0.01; ***p < 0.001; ns, no significant difference was observed.

**Fig 8:**
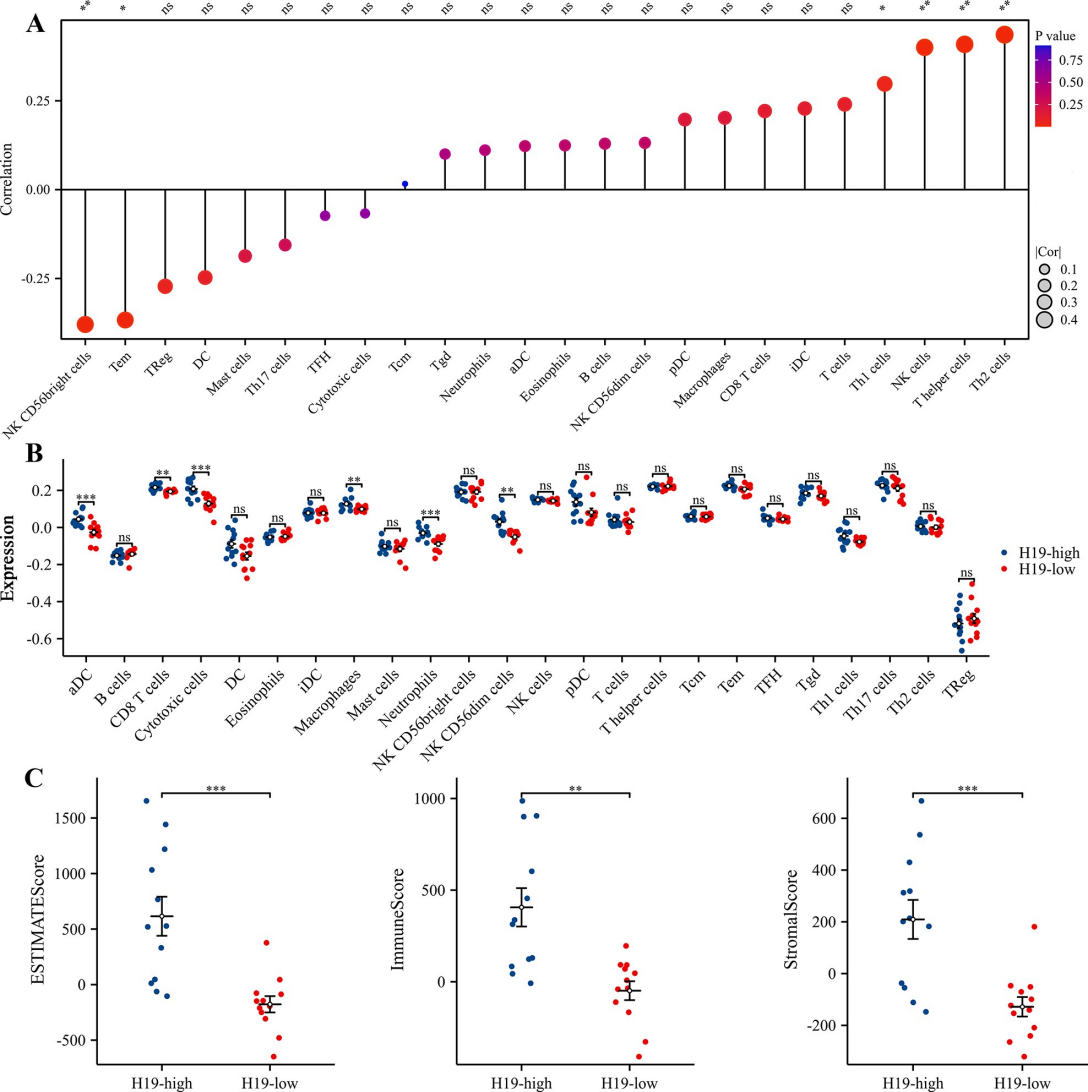
Analysis of H19 expression and its correlation with immune cell infiltration. (A) Correlation analysis between immune cell types and the expression level of H19. (B), Stratification of H19 expression in RIF into high and low expression group based on median expression levels, followed by an analysis of the differential immune cell infiltration patterns associated with aberrant H19 expression. (C) Comparative evaluation of StromalScore, ImmuneScore, and EstimateScore of RIF across different H19 expression group. *p < 0.05; **p < 0.01; ***p < 0.001; ns, no significant difference was observed.

**Fig 9:**
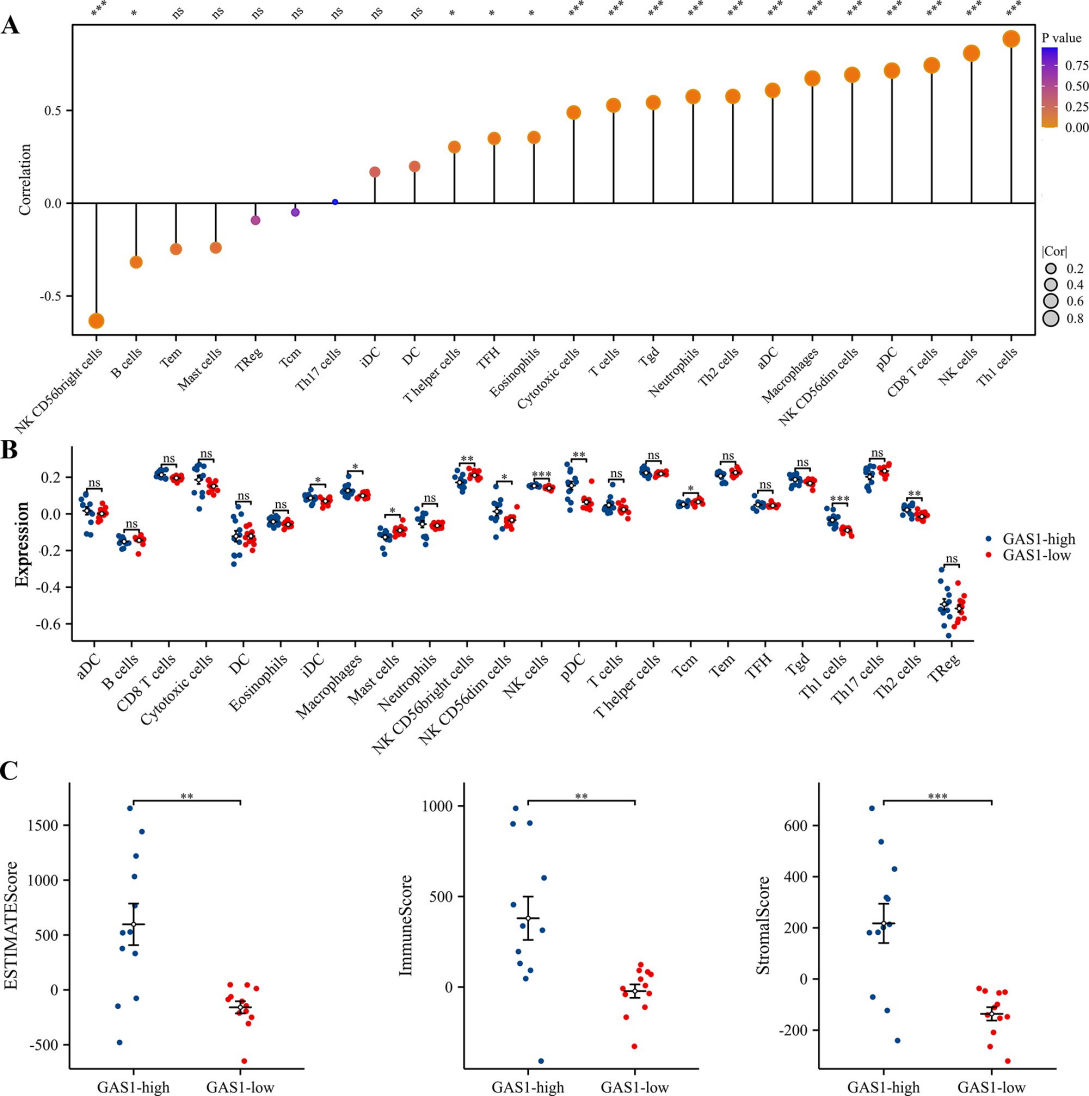
Analysis of GAS1 expression and its correlation with immune cell infiltration in RIF. (A) Correlation analysis illustrating the relationship between various immune cell types and the expression level of GAS1. (B) Categorization of GAS1 expression in RIF into high and low groups based on median expression levels. This is followed by an examination of differing immune cell infiltration patterns associated with varied levels of GAS1 expression. (C) Comparative analysis of StromalScore, ImmuneScore, and EstimateScore in RIF patients, segregated by different H19 expression groups. *p < 0.05; **p < 0.01; ***p < 0.001; ns, no significant difference was observed.

To understand the differences in immune cell composition between RIF and fertile patients, we used CIBERSORTx to deconvolve the bulk RNA sequencing data. CIBERSORT analysis revealed that, compared to the fertile patients, the abundance of T cells gamma delta was statistically lower in patients with RIF (Fig 7A). However, ssGSEA identified 24 immune cell subtypes, including activated CD8 T cells, macrophages, NK CD56dim cells, NK cells, pDC, T cells, T helper cells, Th1 cells, and Th2 cells. These subtypes exhibited lower expression levels of their cell-specific marker genes in the RIF group (Fig 7B). Conversely, the specific marker genes for two immune cell types, NK CD56bright cells and Tem, were expressed at higher levels in the RIF group. Moreover, using the ESTIMATE algorithm, we calculated the StromalScore, ImmuneScore, and EstimateScore for each endometrial sample. T-tests indicated that the RIF group had significantly lower StromalScore, ImmuneScore, and EstimateScore compared to the normal endometrial group (Fig 7C). Additionally, correlation analyses based on the ssGSEA algorithm between H19, GAS1, and 24 immune cells revealed that H19 expression was significantly positively correlated with Th1 cells, NK cells, T helper cells, and Th2 cells, and negatively correlated with NK CD56bright cells and Tem (Fig 8A). GAS1 expression showed significant positive correlations with T helper cells, TFH, eosinophils, cytotoxic cells, T cells, Tgd, Neutrophils, Th2 cells, aDC, Macrophages, NK CD56dim cells, pDC, CD8 T cells, Th1 cells, and NK cells, and negative correlations with NK CD56bright cells and B cells (Fig 9A). Furthermore, based on the median expression values of H19 and GAS1 genes, samples were divided into high and low expression groups. The differences in immune cell infiltration levels between the high and low expression groups were calculated. It was found that the low-expression group of H19 showed significantly lower immune infiltration levels of aDC, CD8 T cells, cytotoxic cells, Macrophages, and Neutrophils compared to the high-expression group (Fig 8B). In the low-expression group of GAS1, iDC, Macrophages, NK CD56dim cells, NK cells, pDC, Th1 cells, and Th2 cells showed significantly lower immune infiltration levels compared to the high-expression group, whereas Mast cells, NK CD56bright cells, and Tcm showed significantly higher levels (Fig 9B). Notably, both low-expression groups of H19 and GAS1 exhibited lower StromalScore, ImmuneScore, and EstimateScore compared to the high-expression groups (Figs 8C and 9C). These findings suggest that abnormally low expression of the H19/GAS1 axis is involved in altering the RIF immune microenvironment.

### Functional role of GAS1 in RIF: Enrichment analysis

**Fig 10:**
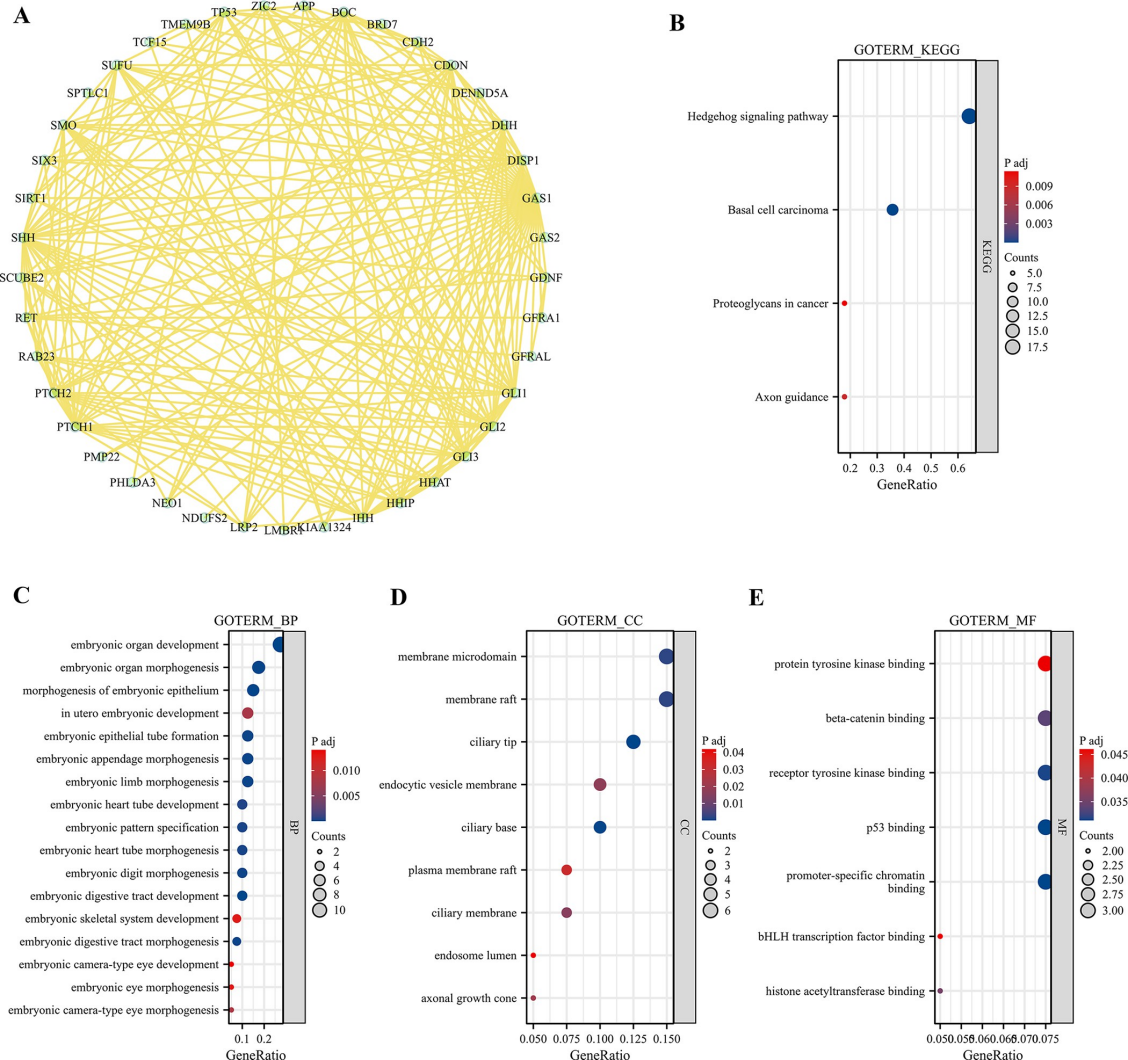
Enrichment analysis of GAS1-related gene. (A) Identification of 41 GAS1-interacting proteins using STRING tool. (B) KEGG pathway analysis conducted on genes interacting with GAS1, highlighting key biological pathways influenced by GAS1 interactions. (C-E) Gene Ontology (GO) enrichment analysis for GAS1 associated genes in RIF, categorizing the function implications of these genes into three aspects: BP (biological process), CC (cellular component), MF (molecular function), thereby elucidating the multifaced roles of GAS1 in cellular and molecular processes.

To further screen out the targeted GAS1-binding proteins and investigate the molecular mechanism of GAS1 in RIF, we acquired 40 GAS1-binding proteins (medium confidence) in the STRING database. Fig 10A shows the protein–protein interaction network. The KEGG pathway enrichment analysis indicated that the hedgehog signaling pathway was most significantly involved in the effect of GAS1 on RIF (Fig 10B). Moreover, GO enrichment analyses, including biological process (Fig 10C), cellular component (Fig 10D), and molecular function (Fig 10E), related to GAS1 were mainly enriched in “in utero embryonic development,” “membrane microdomain,” and “protein tyrosine kinase binding.”

## Discussion

Endometrial receptivity within the window of implantation (WOI) is crucial for successful pregnancy, as it provides an optimal environment for embryo implantation. Disruptions or dysregulations in gene expression within this vital period can significantly affect the development of the endometrium and its complex interaction with the embryo [27]. In line with earlier studies [3, 4, 6, 28], our findings also reveal an aberrantly low expression of key genes such as TP53, leukemia inhibitor factor, vascular endothelial growth factor and human leukocyte antigen (including HLA-A, HLA-F, and HLA-J) in the context of RIF (results are not shown), further underscoring the complexity of the molecular mechanisms at play. Although the role of ceRNAs in regulating cellular processes such as proliferation, apoptosis, migration, and invasion is well-documented in non-tumor female reproductive disorders like polycystic ovary syndrome and endometriosis [20], their presence and influence in the human endometrium, particularly concerning implantation, remain largely unexplored. Our study advances the understanding of Recurrent Implantation Failure (RIF) by highlighting the ceRNA network’s role, specifically the H19-hsa-miR-301a-3p-GAS1 axis, within the endometrium during the window of implantation. This network’s low expression in RIF cases suggests a novel mechanistic insight into endometrial receptivity, pointing towards a disruption in the molecular dialogue essential for successful implantation.

In this investigation, we constructed a lncRNA-miRNA-mRNA triple regulatory network through in silico analysis, encompassing 6 lncRNAs, 8 miRNAs, and 79 mRNAs. Enrichment analysis highlighted the network’s involvement in crucial pathways like cell cycle, membrane raft, and hippo signaling pathway, essential for embryo implantation and development. A focused hub analysis identified a significant subset within this network, involving 2 lncRNAs, 8 miRNAs, and 5 mRNAs, pinpointing critical areas for RIF study. This subset showed diagnostic potential for RIF through detailed expression and ROC curve analyses. Additionally, subcellular localization of the key lncRNAs confirmed their relevance, particularly highlighting the H19-hsa-miR-301a-3p-GAS1 axis’s association with RIF. Notably, our findings suggest GAS1’s unique role in the endometrium, presenting new insights for RIF research.

H19, a maternally expressed lncRNA, plays a pivotal role during embryonic development, as evidenced by its high expression in this phase and subsequent downregulation post-birth [29]. Intriguingly, H19 re-emerges in tumor tissues [30]. Research by S. Ghazal et al. [31] has established a correlation between low H19 expression and reduced endometrial receptivity, and it has been observed to be downregulated in embryonic chorion tissues in cases of spontaneous abortion [32]. Further, aberrant methylation at the H19 imprinting control region in spermatozoa, inheritable by the embryo, disrupts H19 expression, potentially leading to post-implantation loss [33]. These findings collectively underscore the critical role of H19 in embryo implantation and the uterus’s capacity to support pregnancy. Our analysis aligns with these findings, indicating a downregulation of H19 in RIF patients. We also noted that H19 targets hsa-miR-301a-3p, which is upregulated in RIF. Concurrently, we observed low expression of GAS1 in RIF cases. GAS1, a unique component of the vertebrate-specific hedgehog pathway, is essential during the WOI [34] and is a target of hsa-miR-301a-3p. Given the previous report of H19 acting as an hsa-miR-301a-3p sponge to mitigate lung injury in sepsis models [35] and the positive correlation we observed between H19 and GAS1 in RIF, we hypothesize that H19 may serve as a ceRNA, regulating GAS1 expression by competitively binding to hsa-miR-301a-3p. However, this hypothesis necessitates further research to elucidate the underlying mechanisms and validate the functional implications of this ceRNA interaction in the context of RIF.

Enhancing pregnancy success rates in patients with RIF relies heavily on the early prediction of the WOI [36]. Despite ongoing research, the multifactorial pathogenesis of RIF remains largely enigmatic. However, several studies have highlighted factors such as hormonal imbalances, maternal immune system irregularities, and specific genetic polymorphisms as contributory to RIF. Historically, histologic endometrial dating was utilized to optimize timing in fertility treatments. Yet, its application in RIF is now questioned due to the challenges in achieving precise endometrial dating [37, 38] and the unreliable differentiation between fertile and infertile couples using out-of-phase biopsy results [39]. Noncoding RNAs have emerged as key players in the pathogenesis of female reproductive disorders [20], presenting themselves as promising candidates for diagnostic biomarkers and therapeutic targets. In our study, we observed moderate diagnostic accuracy for H19 and GAS1 (AUC > 0.7) and excellent performance for hsa-miR-301a-3p (AUC > 0.9) in predicting RIF. The Comparative Toxicogenomics Database (CTD) further supports this, indicating a notable correlation between H19/GAS1 and embryo loss. Utilizing the predictive results obtained, we were able to identify specific compounds that may exert regulatory effects on H19/GAS1 expression. These findings align with prior research demonstrating the predictive capabilities of both tissue and circulating noncoding RNAs in assessing endometrial receptivity [40, 41], thereby reinforcing the potential of noncoding RNAs as robust diagnostic biomarkers.

The communication between the embryo and the endometrial immune microenvironment, crucial for implantation, is indeed a multifaceted process involving a range of molecules and diverse genetic interplays. As outlined by Robertson et al. [42], the endometrial immune system operates as a ’quality control’ mechanism, fostering implantation under optimal conditions while restricting it in suboptimal physiological states. Typically, the late secretory phase sees significant alterations in the local immune cell composition within a healthy endometrium. This phase is marked by a substantial increase in uterine natural killer (uNK) cells, constituting about 70%–80% of total leukocytes, and macrophages making up 30%, while T cells drop to a mere 10% [43]. Contrastingly, our findings in Recurrent Implantation Failure (RIF) cases reveal a decreased proportion of gamma delta T cells, which is notably enriched in the decidua during normal early pregnancy [44]. This along with the differential expression of immune cell markers in RIF implies a deviation from the normal immunological dynamics within the uterine microenvironment. Specifically, the reduced expression of markers for activated CD8 T cells [45] macrophages [46], and various NK cells types [47] might suggest impaired immune surveillance or a subdued immune response in RIF, potentially compromising the endometrium’s capacity to support successful embryo implantation. Intriguingly, our study also notes an upsurge in NK CD56bright cells and T effector memory (Tem) cell markers in RIF. NK CD56bright cells, predominantly known for their regulatory functions and cytokine production rather than cytotoxic activities [48], may indicate a compensatory response or an immune state alteration not conducive to implantation. The augmented presence of Tem cells in RIF, potentially indicative of heightened memory immune responses [49], further complicates this scenario, potentially impeding the implantation process. Crucially, our study reveals significant correlations between H19 and GAS1 expression and specific immune cell subtypes in the RIF context. This suggests a potential direct or indirect influence of H19 and GAS1 on immune cell populations, thereby impacting endometrial receptivity.

The StromalScore, reflecting the abundance of stromal cells, and the ImmuneScore, indicating the level of immune cell infiltration, together with the EstimateScore which integrates both, offer a multifaceted perspective on the intricacies of the endometrial microenvironment [50, 51]. In our study, we observed significantly lower StromalScore, ImmuneScore, and EstimateScore in patients with Recurrent Implantation Failure (RIF) compared to fertile counterparts. This finding underscores a potential impairment in both the structural and functional integrity of the endometrial stroma in RIF cases. Additionally, it suggests a diminished infiltration of specific immune cell subtypes that are crucial for successful embryo implantation. These alterations in the endometrial environment could be critical factors adversely impacting embryo implantation in RIF patients. Furthermore, our data reveal a compelling association between the aberrantly low expression of H19 and GAS1 genes in RIF and the observed reductions in these scores. This correlation points to a possible mechanistic link where H19/GAS1 might influence the composition and functionality of the uterine microenvironment, further elucidating their roles in the pathophysiology of RIF. The diminished expression of these genes could be contributing to an altered immune landscape and stromal dynamics, thereby affecting the endometrial receptivity.

In our quest to unravel the biological functions of GAS1, we delved into a comprehensive analysis using both KEGG and GO enrichment approaches, focusing on proteins that interact with GAS1. Our KEGG analysis yielded intriguing insights, particularly pointing towards the involvement of the ’hedgehog signaling pathway’. This pathway is renowned for its critical role in embryonic development, suggesting a potential link between GAS1’s functionality and its impact on embryo implantation failure. Expanding our analysis to GO enrichment, which encompasses MF, CC, and BP categories, we discovered that the majority of GAS1-binding proteins are intricately associated with vital processes in embryo development. These include but are not limited to cell membrane modification and kinase binding activities. The implication of these findings is profound, as they suggest that GAS1 could be a key player in modulating cellular mechanisms essential for successful embryo development and implantation.

While our study provides significant insights into the mechanisms of RIF, it is important to acknowledge certain limitations. A primary limitation is that the binding affinities of lncRNAs, miRNAs, and mRNAs, as inferred from the GEO database, necessitate further empirical validation. The predicted pathways and functions, particularly pertaining to the H19/GAS1 axis in the context of RIF, require robust confirmation and detailed exploration through in vitro studies.

## Conclusion

In conclusion, unraveling the intricacies of RIF remains a focal challenge in reproductive medicine. Our research highlights that the dysregulation of the ceRNA network, specifically the H19-hsa-miR-301a-3p-GAS1 axis, may contribute to conditions unfavorable for embryo implantation. This discovery holds significant potential as a diagnostic biomarker for predicting RIF. The insights gained from our study enhance our understanding of RIF’s underlying mechanisms, potentially leading to improved implantation success rates in clinical practice.

## Supporting information

S1 Raw data Raw data and R scripts used for data analysis in this study. (XLSX)

## References

[1] Gnoth C, Maxrath B, Skonieczny T, Friol K, Godehardt E, Tigges J. Final ART success rates: a 10 years survey. Human reproduction (Oxford, England). 2011;26(8):2239–46. Epub 2011/06/11. doi: 10.1093/humrep/der178 . 21659314

[2] Franasiak JM, Alecsandru D, Forman EJ, Gemmell LC, Goldberg JM, Llarena N, et al. A review of the pathophysiology of recurrent implantation failure. Fertility and sterility. 2021;116(6):1436–48. Epub 2021/10/23. doi: 10.1016/j.fertnstert.2021.09.014 . 34674825

[3] Matsuo M, Hirota Y, Fukui Y, Fujita H, Saito-Fujita T, Kaku T, et al. Levonorgestrel Inhibits Embryo Attachment by Eliminating Uterine Induction of Leukemia Inhibitory Factor. Endocrinology. 2020;161(2). Epub 2019/10/23. doi: 10.1210/endocr/bqz005 . 31638694

[4] Lledo B, Turienzo A, Ortiz JA, Morales R, Ten J, Llácer J, et al. Negative effect of P72 polymorphism on p53 gene in IVF outcome in patients with repeated implantation failure and pregnancy loss. J Assist Reprod Genet. 2014;31(2):169–72. Epub 20131211. doi: 10.1007/s10815-013-0147-2 ; PubMed Central PMCID: PMC3933598. 24327010 PMC3933598

[5] Fan W, Huang Z, Li S, Xiao Z. The HLA-G 14-bp polymorphism and recurrent implantation failure: a meta-analysis. J Assist Reprod Genet. 2017;34(11):1559–65. Epub 20170713. doi: 10.1007/s10815-017-0994-3 ; PubMed Central PMCID: PMC5700002. 28707147 PMC5700002

[6] Guo X, Yi H, Li TC, Wang Y, Wang H, Chen X. Role of Vascular Endothelial Growth Factor (VEGF) in Human Embryo Implantation: Clinical Implications. Biomolecules. 2021;11(2). Epub 20210210. doi: 10.3390/biom11020253 ; PubMed Central PMCID: PMC7916576. 33578823 PMC7916576

[7] Zhang D, Yu Y, Duan T, Zhou Q. The role of macrophages in reproductive-related diseases. Heliyon. 2022;8(11):e11686. Epub 20221121. doi: 10.1016/j.heliyon.2022.e11686 ; PubMed Central PMCID: PMC9713353. 36468108 PMC9713353

[8] Von Woon E, Greer O, Shah N, Nikolaou D, Johnson M, Male V. Number and function of uterine natural killer cells in recurrent miscarriage and implantation failure: a systematic review and meta-analysis. Hum Reprod Update. 2022;28(4):548–82. doi: 10.1093/humupd/dmac006 ; PubMed Central PMCID: PMC9247428. 35265977 PMC9247428

[9] Nenonen H, Kondic A, Henic E, Hjelmér I. Recurrent implantation failure and inflammatory markers in serum and follicle fluid of women undergoing assisted reproduction. J Reprod Immunol. 2024;162:104209. Epub 20240130. doi: 10.1016/j.jri.2024.104209 . 38310681

[10] Schumacher A, Sharkey DJ, Robertson SA, Zenclussen AC. Immune Cells at the Fetomaternal Interface: How the Microenvironment Modulates Immune Cells To Foster Fetal Development. J Immunol. 2018;201(2):325–34. doi: 10.4049/jimmunol.1800058 . 29987001

[11] Cech TR, Steitz JA. The noncoding RNA revolution-trashing old rules to forge new ones. Cell. 2014;157(1):77–94. Epub 2014/04/01. doi: 10.1016/j.cell.2014.03.008 . 24679528

[12] Bartel DP. MicroRNAs: target recognition and regulatory functions. Cell. 2009;136(2):215–33. Epub 2009/01/27. doi: 10.1016/j.cell.2009.01.002 ; PubMed Central PMCID: PMC3794896. 19167326 PMC3794896

[13] Lewis BP, Burge CB, Bartel DP. Conserved seed pairing, often flanked by adenosines, indicates that thousands of human genes are microRNA targets. Cell. 2005;120(1):15–20. Epub 2005/01/18. doi: 10.1016/j.cell.2004.12.035 . 15652477

[14] Chen CH, Lu F, Yang WJ, Yang PE, Chen WM, Kang ST, et al. A novel platform for discovery of differentially expressed microRNAs in patients with repeated implantation failure. Fertility and sterility. 2021;116(1):181–8. Epub 2021/04/08. doi: 10.1016/j.fertnstert.2021.01.055 . 33823989

[15] Rah H, Chung KW, Ko KH, Kim ES, Kim JO, Sakong JH, et al. miR-27a and miR-449b polymorphisms associated with a risk of idiopathic recurrent pregnancy loss. PloS one. 2017;12(5):e0177160. Epub 2017/05/11. doi: 10.1371/journal.pone.0177160 ; PubMed Central PMCID: PMC5425187. 28489914 PMC5425187

[16] Esmaeilivand M, Abedelahi A, Hamdi K, Farzadi L, Goharitaban S, Fattahi A, et al. Role of miRNAs in preimplantation embryo development and their potential as embryo selection biomarkers. Reproduction, fertility, and development. 2022;34(8):589–97. Epub 2022/04/21. doi: 10.1071/RD21274 . 35440361

[17] Liang J, Wang S, Wang Z. Role of microRNAs in embryo implantation. Reproductive biology and endocrinology: RB&E. 2017;15(1):90. Epub 2017/11/23. doi: 10.1186/s12958-017-0309-7 ; PubMed Central PMCID: PMC5699189. 29162091 PMC5699189

[18] Wang C, Wang L, Ding Y, Lu X, Zhang G, Yang J, et al. LncRNA Structural Characteristics in Epigenetic Regulation. International journal of molecular sciences. 2017;18(12). Epub 2018/01/03. doi: 10.3390/ijms18122659 ; PubMed Central PMCID: PMC5751261. 29292750 PMC5751261

[19] Salmena L, Poliseno L, Tay Y, Kats L, Pandolfi PP. A ceRNA hypothesis: the Rosetta Stone of a hidden RNA language? Cell. 2011;146(3):353–8. Epub 20110728. doi: 10.1016/j.cell.2011.07.014 ; PubMed Central PMCID: PMC3235919. 21802130 PMC3235919

[20] Yang Y, Xiong Y, Pan Z. Role of ceRNAs in non-tumor female reproductive diseases†. Biol Reprod. 2023;108(3):363–81. Epub 2022/11/11. doi: 10.1093/biolre/ioac200 . 36355359

[21] Gu Z, Eils R, Schlesner M. Complex heatmaps reveal patterns and correlations in multidimensional genomic data. Bioinformatics. 2016;32(18):2847–9. doi: 10.1093/bioinformatics/btw313 27207943

[22] Hsu SD, Lin FM, Wu WY, Liang C, Huang WC, Chan WL, et al. miRTarBase: a database curates experimentally validated microRNA-target interactions. Nucleic Acids Res. 2011;39(Database issue):D163–9. Epub 20101110. doi: 10.1093/nar/gkq1107 ; PubMed Central PMCID: PMC3013699. 21071411 PMC3013699

[23] Vlachos IS, Paraskevopoulou MD, Karagkouni D, Georgakilas G, Vergoulis T, Kanellos I, et al. DIANA-TarBase v7.0: indexing more than half a million experimentally supported miRNA:mRNA interactions. Nucleic Acids Res. 2015;43(Database issue):D153–9. Epub 20141121. doi: 10.1093/nar/gku1215 ; PubMed Central PMCID: PMC4383989. 25416803 PMC4383989

[24] Xiao F, Zuo Z, Cai G, Kang S, Gao X, Li T. miRecords: an integrated resource for microRNA-target interactions. Nucleic Acids Res. 2009;37(Database issue):D105–10. Epub 20081107. doi: 10.1093/nar/gkn851 ; PubMed Central PMCID: PMC2686554. 18996891 PMC2686554

[25] Bindea G, Mlecnik B, Tosolini M, Kirilovsky A, Waldner M, Obenauf AC, et al. Spatiotemporal dynamics of intratumoral immune cells reveal the immune landscape in human cancer. Immunity. 2013;39(4):782–95. doi: 10.1016/j.immuni.2013.10.003 . 24138885

[26] Yoshihara K, Shahmoradgoli M, Martínez E, Vegesna R, Kim H, Torres-Garcia W, et al. Inferring tumour purity and stromal and immune cell admixture from expression data. Nat Commun. 2013;4:2612. doi: 10.1038/ncomms3612 ; PubMed Central PMCID: PMC3826632. 24113773 PMC3826632

[27] Kao LC, Tulac S, Lobo S, Imani B, Yang JP, Germeyer A, et al. Global gene profiling in human endometrium during the window of implantation. Endocrinology. 2002;143(6):2119–38. doi: 10.1210/endo.143.6.8885 . 12021176

[28] Nilsson LL, Hviid TVF. HLA Class Ib-receptor interactions during embryo implantation and early pregnancy. Hum Reprod Update. 2022;28(3):435–54. doi: 10.1093/humupd/dmac007 . 35234898

[29] Jiang X, Ning Q. The mechanism of lncRNA H19 in fibrosis and its potential as novel therapeutic target. Mech Ageing Dev. 2020;188:111243. Epub 20200413. doi: 10.1016/j.mad.2020.111243 . 32298666

[30] Ghafouri-Fard S, Esmaeili M, Taheri M. H19 lncRNA: Roles in tumorigenesis. Biomed Pharmacother. 2020;123:109774. Epub 20191225. doi: 10.1016/j.biopha.2019.109774 . 31855739

[31] Ghazal S, McKinnon B, Zhou J, Mueller M, Men Y, Yang L, et al. H19 lncRNA alters stromal cell growth via IGF signaling in the endometrium of women with endometriosis. EMBO Mol Med. 2015;7(8):996–1003. doi: 10.15252/emmm.201505245 ; PubMed Central PMCID: PMC4551339. 26089099 PMC4551339

[32] He D, Zeng H, Chen J, Xiao L, Zhao Y, Liu N. H19 regulates trophoblastic spheroid adhesion by competitively binding to let-7. Reproduction. 2019;157(5):423–30. doi: 10.1530/REP-18-0339 ; PubMed Central PMCID: PMC6433002. 30780128 PMC6433002

[33] Doshi T, D’Souza C, Vanage G. Aberrant DNA methylation at Igf2-H19 imprinting control region in spermatozoa upon neonatal exposure to bisphenol A and its association with post implantation loss. Mol Biol Rep. 2013;40(8):4747–57. Epub 20130508. doi: 10.1007/s11033-013-2571-x . 23653003

[34] Tan Q, Shi S, Liang J, Zhang X, Cao D, Wang Z. MicroRNAs in Small Extracellular Vesicles Indicate Successful Embryo Implantation during Early Pregnancy. Cells. 2020;9(3). Epub 20200306. doi: 10.3390/cells9030645 ; PubMed Central PMCID: PMC7140406. 32155950 PMC7140406

[35] Hou J, He M, Chen Q, Liang S. LncRNA H19 acts as miR-301a-3p sponge to alleviate lung injury in mice with sepsis by regulating Adcy1. Immunopharmacol Immunotoxicol. 2022;44(4):565–73. Epub 20220427. doi: 10.1080/08923973.2022.2067045 . 35438054

[36] Enciso M, Aizpurua J, Rodríguez-Estrada B, Jurado I, Ferrández-Rives M, Rodríguez E, et al. The precise determination of the window of implantation significantly improves ART outcomes. Sci Rep. 2021;11(1):13420. Epub 20210628. doi: 10.1038/s41598-021-92955-w ; PubMed Central PMCID: PMC8238935. 34183760 PMC8238935

[37] Smith S, Hosid S, Scott L. Endometrial biopsy dating. Interobserver variation and its impact on clinical practice. J Reprod Med. 1995;40(1):1–3. . 7722968

[38] Deligdisch L. Hormonal pathology of the endometrium. Mod Pathol. 2000;13(3):285–94. doi: 10.1038/modpathol.3880050 . 10757339

[39] Coutifaris C, Myers ER, Guzick DS, Diamond MP, Carson SA, Legro RS, et al. Histological dating of timed endometrial biopsy tissue is not related to fertility status. Fertil Steril. 2004;82(5):1264–72. doi: 10.1016/j.fertnstert.2004.03.069 . 15533340

[40] Panir K, Schjenken JE, Robertson SA, Hull ML. Non-coding RNAs in endometriosis: a narrative review. Hum Reprod Update. 2018;24(4):497–515. doi: 10.1093/humupd/dmy014 . 29697794

[41] Hull ML, Nisenblat V. Tissue and circulating microRNA influence reproductive function in endometrial disease. Reprod Biomed Online. 2013;27(5):515–29. Epub 20130827. doi: 10.1016/j.rbmo.2013.07.012 . 24055529

[42] Robertson SA, Moldenhauer LM, Green ES, Care AS, Hull ML. Immune determinants of endometrial receptivity: a biological perspective. Fertil Steril. 2022;117(6):1107–20. doi: 10.1016/j.fertnstert.2022.04.023 . 35618356

[43] Alecsandru D, Klimczak AM, Garcia Velasco JA, Pirtea P, Franasiak JM. Immunologic causes and thrombophilia in recurrent pregnancy loss. Fertil Steril. 2021;115(3):561–6. Epub 20210217. doi: 10.1016/j.fertnstert.2021.01.017 . 33610320

[44] Yang S, Feng T, Ma C, Wang T, Chen H, Li L, et al. Early pregnancy human decidua gamma/delta T cells exhibit tissue resident and specific functional characteristics. Mol Hum Reprod. 2022;28(8). doi: 10.1093/molehr/gaac023 . 35758607

[45] Cabarrocas J, Bauer J, Piaggio E, Liblau R, Lassmann H. Effective and selective immune surveillance of the brain by MHC class I-restricted cytotoxic T lymphocytes. Eur J Immunol. 2003;33(5):1174–82. doi: 10.1002/eji.200323492 . 12731042

[46] Tang PM, Nikolic-Paterson DJ, Lan HY. Macrophages: versatile players in renal inflammation and fibrosis. Nat Rev Nephrol. 2019;15(3):144–58. Epub 20190128. doi: 10.1038/s41581-019-0110-2 . 30692665

[47] Pyz E, Marshall AS, Gordon S, Brown GD. C-type lectin-like receptors on myeloid cells. Ann Med. 2006;38(4):242–51. doi: 10.1080/07853890600608985 . 16754255

[48] Keating SE, Zaiatz-Bittencourt V, Loftus RM, Keane C, Brennan K, Finlay DK, et al. Metabolic Reprogramming Supports IFN-γ Production by CD56bright NK Cells. J Immunol. 2016;196(6):2552–60. Epub 20160212. doi: 10.4049/jimmunol.1501783 . 26873994

[49] Ando M, Ito M, Srirat T, Kondo T, Yoshimura A. Memory T cell, exhaustion, and tumor immunity. Immunol Med. 2020;43(1):1–9. Epub 20191210. doi: 10.1080/25785826.2019.1698261 . 31822213

[50] Wang J, Shi W, Miao Y, Gan J, Guan Q, Ran J. Evaluation of tumor microenvironmental immune regulation and prognostic in lung adenocarcinoma from the perspective of purinergic receptor P2Y13. Bioengineered. 2021;12(1):6286–304. doi: 10.1080/21655979.2021.1971029 ; PubMed Central PMCID: PMC8806861. 34494914 PMC8806861

[51] Sui S, An X, Xu C, Li Z, Hua Y, Huang G, et al. An immune cell infiltration-based immune score model predicts prognosis and chemotherapy effects in breast cancer. Theranostics. 2020;10(26):11938–49. Epub 20201025. doi: 10.7150/thno.49451 ; PubMed Central PMCID: PMC7667685. 33204321 PMC7667685

